# Distribution based nearest neighbor imputation for truncated high dimensional data with applications to pre-clinical and clinical metabolomics studies

**DOI:** 10.1186/s12859-017-1547-6

**Published:** 2017-02-20

**Authors:** Jasmit S. Shah, Shesh N. Rai, Andrew P. DeFilippis, Bradford G. Hill, Aruni Bhatnagar, Guy N. Brock

**Affiliations:** 1 Department of Bioinformatics and Biostatistics, University of Louisville, Louisville, KY 40202 USA; 2 Department of Medicine, Division of Cardiovascular Medicine, Diabetes and Obesity Center, University of Louisville, Louisville, KY 40202 USA; 3 Present Affiliation: Department of Biomedical Informatics, The Ohio State University, Columbus, OH 43210 USA

**Keywords:** Metabolomics, Missing value, Imputation, Truncated normal, High dimensional data, K-nearest neighbors

## Abstract

**Background:**

High throughput metabolomics makes it possible to measure the relative abundances of numerous metabolites in biological samples, which is useful to many areas of biomedical research. However, missing values (MVs) in metabolomics datasets are common and can arise due to both technical and biological reasons. Typically, such MVs are substituted by a minimum value, which may lead to different results in downstream analyses.

**Results:**

Here we present a modified version of the K-nearest neighbor (KNN) approach which accounts for truncation at the minimum value, i.e., KNN truncation (KNN-TN). We compare imputation results based on KNN-TN with results from other KNN approaches such as KNN based on correlation (KNN-CR) and KNN based on Euclidean distance (KNN-EU). Our approach assumes that the data follow a truncated normal distribution with the truncation point at the detection limit (LOD). The effectiveness of each approach was analyzed by the root mean square error (RMSE) measure as well as the metabolite list concordance index (MLCI) for influence on downstream statistical testing. Through extensive simulation studies and application to three real data sets, we show that KNN-TN has lower RMSE values compared to the other two KNN procedures as well as simpler imputation methods based on substituting missing values with the metabolite mean, zero values, or the LOD. MLCI values between KNN-TN and KNN-EU were roughly equivalent, and superior to the other four methods in most cases.

**Conclusion:**

Our findings demonstrate that KNN-TN generally has improved performance in imputing the missing values of the different datasets compared to KNN-CR and KNN-EU when there is missingness due to missing at random combined with an LOD. The results shown in this study are in the field of metabolomics but this method could be applicable with any high throughput technology which has missing due to LOD.

**Electronic supplementary material:**

The online version of this article (doi:10.1186/s12859-017-1547-6) contains supplementary material, which is available to authorized users.

## Background

High throughput technology makes it possible to generate high dimensional data in many areas of biochemical research. Mass spectrometry (MS) is one of the important high-throughput analytical techniques used for profiling small molecular compounds, such as metabolites, in biological samples. Raw data from a metabolomics experiment usually consist of the retention time (if liquid or gas chromatography is used for separation), the observed mass to charge ratio, and a measure of ion intensity [1]. The ion intensity represents the measure of each metabolite’s relative abundance whereas the mass-to-charge ratios and the retention times assist in identifying unique metabolites. A detailed pre-processing of the raw data, including baseline correction, noise reduction, smoothing, peak detection and alignment and peak integration, is necessary before analysis [2]. The end product of this processing step is a data matrix consisting of the unique features and its intensity measures in each sample. Commonly, data generated from MS have many missing values. Missing values (MVs) in MS can occur from various sources both technical and biological. There are three common sources of missingness: [1] i) a metabolite could be truly missing from a sample due to biological reasons, ii) a metabolite can be present in a sample but at a concentration below the detection limit of the MS, and iii) a metabolite can be present in a sample at a level above the detection limit but fail to be detected due to technical issues related to sample processing.

The limit of detection (LOD) is the smallest sample quantity that yields a signal that can be differentiated from the background noise. Shrivastava et al. [3] give different guidelines for the detection limit and describe different methods for calculating the detection limit. Some common methods [3] for the estimation of detection limits are visual definition, calculation from signal to noise ratio, calculation from standard deviation (SD) of the blanks and calculation from the calibration line at low concentrations. Armbruster et al. [4] compare the empirical and statistical methods based on gas chromatography MS assays for drugs. They explain the calculation from SD where a series of blank (negative) samples (a sample containing no analyte but with a matrix identical to that of the average sample analyzed) are tested and the mean blank value and the SD are calculated, where the LOD is the mean blank value plus two or three SDs [4]. The signal-to-noise ratio method is commonly applied to analytical methods that exhibit baseline noise [3, 5]. In this method, the peak-to-peak noise around the analyte retention time is measured, and subsequently, the concentration of the analyte that would yield a signal equal to a signal-to-noise ratio (S/N) of three is generally accepted for estimating the LOD [3].

Missing data can be classified into three categories based on the properties of the causality of the missingness [6]: “missing completely at random (MCAR)”, “missing at random (MAR)” and “missing not at random (MNAR)”. The missing values are considered MCAR if the probability of an observation being missing does not depend on observed or unobserved measurements. If the probability of an observation being missing depends only on observed measurements then the values are considered as MAR. MNAR is when the probability of an observation being missing depends on unobserved measurements. In metabolomics studies, we assume that the missing values occurs either as MNAR (metabolites occur at low abundances, below the detection limit) or MAR, e.g., metabolites are truly not present or are above the detection limit but missing due to technical errors. The majority of imputation algorithms for high-throughput data exploit the MAR mechanism and use observed values from other genes/proteins/metabolites to impute the MVs. However, imputation for MNAR values is fraught with difficulty [1, 7]. Using the imputation methods for microarray studies in MS omics studies could lead to biased results because most of the imputation techniques produce unbiased results only if the missing data are MCAR or MAR [8]. Karpievitch et al. [7] discuss several approaches in dealing with missing values, considering MNAR as censored in proteomic studies.

Many statistical analyses require a complete dataset and therefore missing values are commonly substituted with a reliable value. Many MV imputation methods have been developed in the literature in other -omic studies. For example the significance of appropriate handling of MVs has been acknowledged in the analysis of DNA microarray [9] and gel based proteomics data [10, 11]. Brock et al. [12] evaluated a variety of imputation algorithms with expression data such as KNN, singular value decomposition, partial least squares, Bayesian principal component analysis, local least squares and least squares adaptive. In MS data analysis, a common approach is to drop individual metabolites with a large proportion of subjects with missing values from the analysis or to drop the entire subject with a large number of missing metabolites. Other standard methods of substitution include using a minimum value, mean, or median value. Gromski et al. [13] analyzed different MV imputation methods and their influence on multivariate analysis. The choice of imputation method can significantly affect the results and interpretation of analyses of metabolomics data [14].

**Fig. 1:**
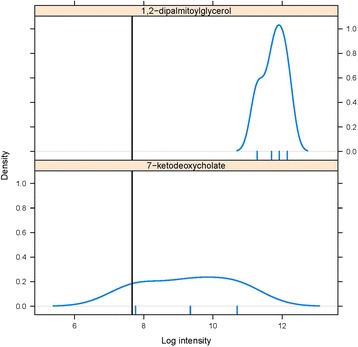
Two examples of metabolite distributions which have missing values (MVs), from the myocardial infarction data [15]. The *black vertical line* on each graph shows the minimum value of the data, considered as the lower limit of detection (LOD). The *small vertical lines* below the x-axis in each case indicate the observed values of the metabolites. The figure on the top shows the distribution of 1,2 dipalmitoylglycerol, where the observed values are all around three standard deviations above the LOD. In this case, the MVs are likely to be MAR or MCAR. In contrast, the figure on the bottom shows the distribution of 7-ketodeoxycholate, which is close to the LOD. Here, the MVs are likely to be below the LOD and hence MNAR

Since missingness may be due to a metabolite being below the detection limit of the mass spectrometer (MNAR) or other technical issues unrelated to the abundance of the metabolite (MAR), we develop a method that accounts for both of these mechanisms. To demonstrate missing patterns, Fig. 1 summarizes the distribution of two different metabolites taken from Sansbury et al. [15], both of which had missing values. The top graph shows that the distribution of the metabolite is far above the detection limit and therefore replacing the MV in that metabolite with a LOD value would be inappropriate. Similarly, the bottom graph shows that the distribution of the metabolite is near the detection limit and therefore replacing the MV with a mean or median value might be inappropriate.

In this work, we develop an imputation algorithm based on nearest neighbors that considers MNAR and MAR together based on a truncated distribution, with the detection limit considered as the truncation point. The proposed truncation-based KNN method is compared to standard KNN imputation based on Euclidean and correlation based distance metrics. We show that this method is effective and generally outperforms the other two KNN procedures through extensive simulation studies and application to three real data sets [15, 16].

## Methods

### K-Nearest Neighbors (NN)

KNN is a non-parametric machine learning algorithm. NN imputation approaches are neighbor based methods where the imputed value is either a value that was measured for the neighbor or the average of measured values for multiple neighbors. It is a very simple and powerful method. The motivation behind the NN algorithm is that samples with similar features have similar output values. The algorithm works on the premise that the imputation of the unknown samples can be done by relating the unknown to the known according to some distance or similarity function. Essentially, two vectors that are far apart based on the distance function are less likely than two closely situated vectors to have a similar output value. The most frequently used distance metrics are the Euclidean distance metric or the Pearson correlation metric. Let *X*_*i*_, *i* = 1, …, *n* be independent and identically distributed (iid) with mean *µ*_*X*_ and standard deviation *σ*_*X*_, and *Y*_*i*_, *i* = 1, …, *n* be iid with mean *µ*_*Y*_ and standard deviation *σ*_*Y*_. The two sets of measurements are assumed to be taken on the same set of observations. Then the Euclidean distance between the two sample vectors ***x*** = *x*_1_, …, *x*_*n*_ and ***y*** = *y*_1_, …, *y*_*n*_ is defined as follows:

$$ {d}_E\left(\boldsymbol{x},\boldsymbol{y}\right) = \sqrt{{\displaystyle {\sum}_{i=1}^n{\left({x}_i-{y}_i\right)}^2}} $$

It is the ordinary distance between two points in the Euclidean space. The correlation between vectors ***x*** and ***y*** is defined as follows:

$$ r\left(\boldsymbol{x},\boldsymbol{y}\right) = \frac{\frac{1}{n}{\displaystyle {\sum}_i}{x}_i{y}_i{\widehat{\mu}}_X{\widehat{\mu}}_Y}{{\widehat{\sigma}}_X{\widehat{\sigma}}_Y} $$

where ${\widehat{\mu}}_X$, ${\widehat{\mu}}_Y$, ${\widehat{\sigma}}_X,$ and ${\widehat{\sigma}}_Y$ are the sample estimates of the corresponding population parameters. If ***x*** and ***y*** are standardized (denoted as ***x*** ^*s*^ and ***y*** ^*s*^, respectively) to each have a mean of zero and a standard deviation of one, the formula reduces to:

$$ r\left({\boldsymbol{x}}^s,{\boldsymbol{y}}^s\right)=\frac{1}{n}{\displaystyle \sum_{i=1}^n}{x}_i{y}_i $$

When using the Euclidean distance, normalization/re-scaling process is not required for KNN imputation because neighbors with similar magnitude to the metabolite with MV are used for imputation. In the correlation based distance, since metabolites can be highly correlated but different in magnitude, the metabolites are first standardized to mean zero and standard deviation one before the neighbor selection and then re-scaled back to the original scale after imputation [12, 17]. The distance used to select the neighbors is *d*_*C*_ = 1 − |*r*|, where *r* is the Pearson correlation. This distance allows for information to be incorporated from both positively correlated and negatively correlated neighbors. During the distance calculation MVs are omitted, so that it is based only on the complete pairwise observations between two metabolites.

The KNN based on the Euclidean (KNN-EU) or Correlation (KNN-CR) distance metrics do not account for the truncation at the minimum value or the limit of detection. In our method, we propose a modified version of the KNN approach which accounts for the truncation at the minimum value called KNN Truncation (KNN-TN). A truncated distribution occurs when there is no ability to know about data that falls below a set threshold or outside a certain set range. Often the general idea is to make inference back to the original population and not on the truncated population and therefore inference is made on the population mean and not the truncated sample mean. In the regular KNN-CR, the metabolites are standardized based on the sample mean and sample standard deviation. In KNN-TN, we first estimate the means and standard deviation, and use the estimated values for standardizing. Maximum likelihood Estimators (MLE) are estimated for the truncated normal distribution. The likelihood for the truncated normal distribution is

$$ L\left(\mu, {\sigma}^2\right) = {\displaystyle \prod_{i=1}^n}\left(\frac{1}{P\left( Y\in \left( a,\infty \right)\Big|\mu, {\sigma}^2\right)}\right)\left(\frac{1}{\sqrt{2\pi {\sigma}^2}}\right){e}^{\frac{-{\left({y}_i-\mu \right)}^2}{2{\sigma}^2}} $$

Here *a* is the truncation point and presumed to be known in our case. Also note that MVs are ignored and the likelihood is based only on the observed data (in essence a partial likelihood akin to a Cox regression model ([18, 19])). The log likelihood is then

$$ l= \ln L\left(\mu, {\sigma}^2\right) = - n \ln \left( P\left( Y\in \left( a,\infty \right)\Big|\mu, {\sigma}^2\right)\right)- n \ln \left(\sqrt{2\pi {\sigma}^2}\right) - \frac{{\displaystyle \sum }{\left({y}_i-\mu \right)}^2}{2{\sigma}^2} $$

The *P*(*Y* ∈ (*a*, ∞)|*μ*, *σ* ^2^) is the part of the likelihood that is specific to the truncated normal distribution.

We use the Newton–Raphson (NR) optimization procedure to find the MLEs for μ and σ [20, 21] (for details see the Additional file 1). The sample means and standard deviations are used as the initial values for the NR optimization. To accelerate the run-time of the algorithm, truncation-based estimation of the mean and standard deviation was done only on metabolites that had a sample mean within three standard deviations of the LOD. For the other metabolites, we simply used the sample means and standard deviations. The runtime for one dataset with 50 samples and 400 metabolites and the three missing mechanisms was about 1.20 min on average, which included truncation-based estimation of the mean and standard deviation and the three imputation methods. In particular for one individual run on 50 samples and 400 metabolites with 15% missingness, the runtime was about 1.81 s for the KNN-EU method, 3.41 s for the KNN-CR method and 19.95 s for the KNN-TN method. The KNN-TN method runtime was a little longer due to the estimation of the means and standard deviations.

Let *y*_*im*_ be the intensity of metabolite *m* (1 ≤ *m* ≤ *M*) in sample *i* (1 ≤ *i* ≤ *N*). The following steps outline the KNN imputation algorithms (KNN-TN, KNN-CR, and KNN-EU) in our paper:

1. Choose a *K* to use for the number of nearest neighbors.
2. Select the distance metric: Euclidean (KNN-EU) or correlation (KNN-CR and KNN-TN)
3. If using correlation metric, decide whether to standardize the data based on sample mean and sample standard deviation (KNN-CR) or the truncation-based estimate of the mean and standard deviation (KNN-TN).
4. Based on the distance metric and (possibly) standardization, for each metabolite with a missing value in sample *i* find the *K* closest neighboring metabolites which have an observed value in sample *i*.
5. For metabolite *m* with missing value in sample *i*, calculated the imputed value *ŷ*_*im*_ by taking the weighted average of the *K* nearest neighbors for each missing value in the metabolite. The weights are calculated as ${w}_k = \mathrm{sign}\left({r}_k\right){d}_k^{-1}/{\displaystyle \sum_{l=1}^K}{d}_l^{-1}$, where *d*_1_, …, *d*_*K*_ are the distances between metabolite *m* and each of the *K* neighbors and *r*_1_, …, *r*_*K*_ are the corresponding Pearson correlations. The multiplication by sign(*r*_*k*_) allows for incorporation of negatively correlated metabolites. The imputed value is then ${\widehat{y}}_{im} = \frac{1}{K}{\displaystyle \sum_{k=1}^K}{w}_k{y}_{ik}$.
6. If using the KNN-CR or KNN-TN approaches, back-transform into the original space of the metabolites.

**Fig. 2:**
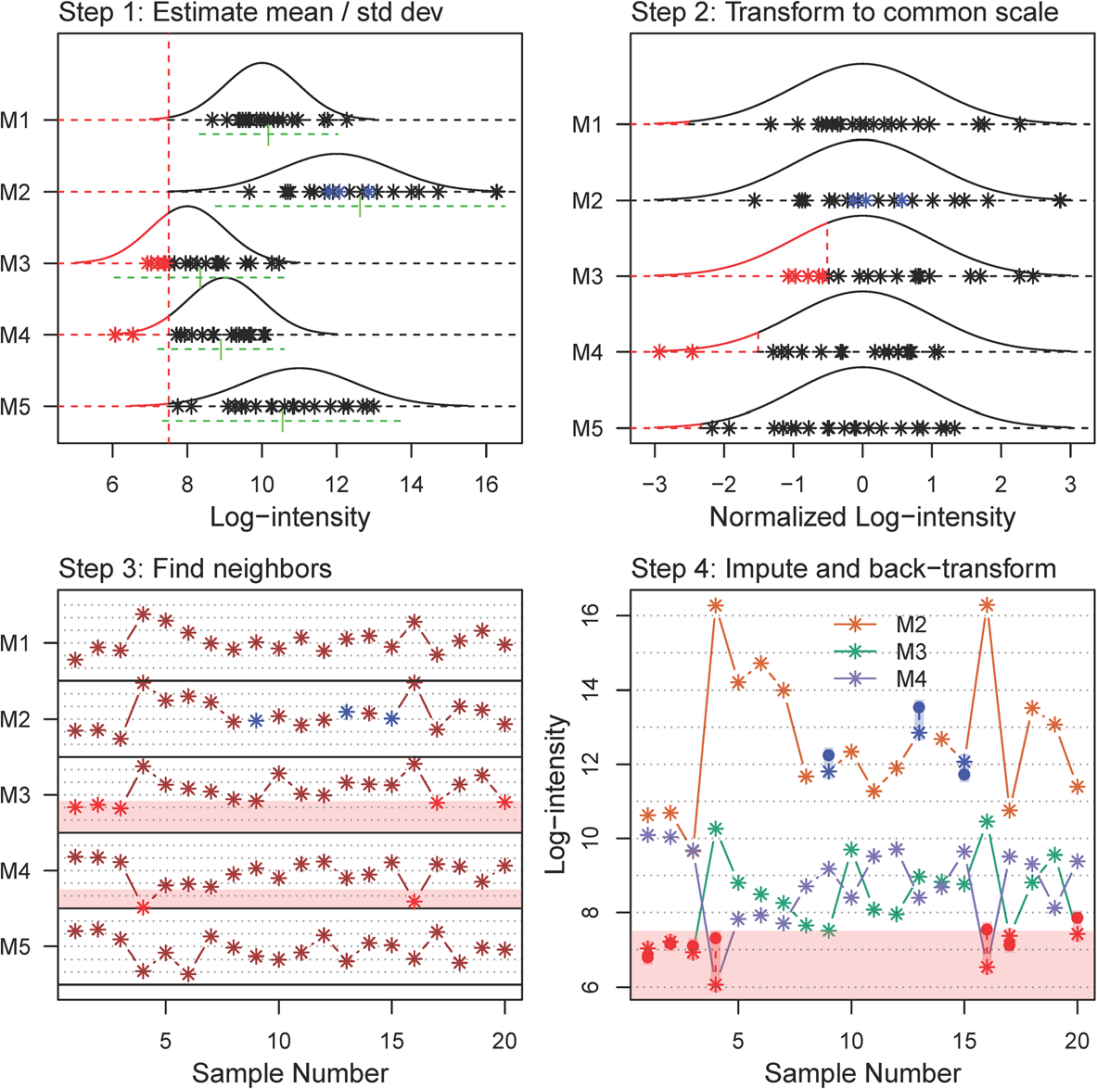
Steps in the KNN-TN imputation algorithm. Step 1 (*top left panel*): The first step in the KNN-TN procedure is to estimate the mean and standard deviation of each metabolite. Here, the distribution and simulated values for five metabolites (M1-M5) and 20 samples are given. For each metabolite, observed values are given by *black stars*. Additionally, M2 has three values that are MAR (*blue stars*), while M3 has five points that are MNAR (below the LOD, *red stars*) and M4 has two points below the LOD (*red stars*). The estimate mean for each metabolite is indicated by the *underlying green vertical dash*, while the *green horizontal dashed line* represents the estimated standard deviation (the line extends out +/− two standard deviations). Step 2 (*top right panel*): The second step in the procedure is to transform all the values to a common scale, with mean zero and standard deviation of one for each metabolite. The original points are represented in this transformed scale with *black stars*, with MNAR values in red and MAR values in blue. Step 3 (*bottom left panel*): The next step is to find metabolites with a similar profile on this common scale. In this case, metabolites M1-M3 are highly correlated and M4-M5 are also highly correlated. The two groups of metabolites are also negatively correlated with each other, and this information can also be used to aid the imputation process. The missing values are imputed in the transformed space, with weights based on the inverse of the distances 1 − |*r*| (*r* is the Pearson correlation between the two metabolites). Contributions from negatively correlated metabolites are multiplied by negative one. The region below the LOD is shaded light red. Step 4 (*bottom right panel*): The values are then back-transformed to the original space based on the estimated means and standard deviations from Step 1. Here, we show the three metabolites with missing values M2, M3, and M4. *Solid circles* represent imputed values for MAR (*blue circles*) and MNAR (*red circles*). The region below the LOD is again shaded in *light red*, while the slightly darker shaded regions connect the imputed value with its underlying true value. The imputed values are fairly close to the true values for metabolites M2 and M3, while for metabolite M4 the values are further away due to under-estimation of the true variance for M4 (c.f. *top left panel*)

The steps for the KNN-TN procedure are outlined graphically in Fig. 2 (see figure caption for detailed explanation). The graph illustrates the algorithms success at imputing both MAR and MNAR values.

#### Assessment of performance

We evaluated the performance of the imputation methods by using the root mean squared error (RMSE) as the metric. It measures the difference between the estimated values and the original true values, when the original true values are known. The following simulation procedure from a complete dataset with no MVs is performed. MVs are generated by removing a proportion *p* of values from the complete data to generate data with MVs. The MVs are then imputed as *ŷ*_*mi*_ using the given imputation method. Finally, the root mean squared error (RMSE) is used to assess the performance by comparing the values of the imputed entries with the true values:

$$ RMSE = \sqrt{\frac{1}{n\left(\mathrm{\mathcal{M}}\right)\ }{\displaystyle \sum_{y_{im}\ \in\ \mathit{\mathcal{M}}}}{\left({\widehat{y}}_{im}-{y}_{im}\right)}^2}, $$

where ℳ is the set of missing values and *n*(ℳ) is the cardinality or number of elements in ℳ. Statistical significance of differences in RMSE values between methods was determined using multi-factor ANOVA models (with pre-defined contrasts for differences between the methods), with main effects for each factor in the simulation study. We further evaluate the biological impact of MV imputation on downstream analysis, specifically analyzing differences in mean log intensity between groups via the *t*-test. We evaluate the performances of the MV imputation using the metabolite list concordance index (MLCI) [22]. By applying a selected MV imputation method, one metabolite list is obtained from the complete data and another is obtained from the imputed data. The MLCI is defined as:

$$ MLCI\ \left({M}_{C D},{M}_{ID}\right) = \frac{n\left({M}_{C D}{\displaystyle \cap }{M}_{ID}\right)}{n\left({M}_{C D}\right)} + \frac{n\left({M}_{C D}^C{\displaystyle \cap }{M}_{ID}^C\right)}{n\left({M}_{C D}^C\right)}-1, $$

where *M*_*CD*_ is the list of statistically significant metabolites in the complete data, *M*_*ID*_ is the list of statistically significant metabolites in the imputed data, and *M*_*CD*_^*C*^ and *M*_*ID*_^*C*^ represent their complements, respectively. The metabolite list taken from the complete dataset is considered as the gold standard and a high value in MLCI indicates that the metabolite list from the imputed data is similar to that from the complete data.

#### Simulation study

We carried out a simulation study to compare the performance of the three different KNN based imputation methods. The simulations were conducted with 100 replications and are similar in spirit to those used in Tutz and Ramzan, 2015 [17]. For each replication we generated data with different combinations of sample sizes *n* and number of metabolites *m*. Each set of metabolites for a given sample were drawn from a *m* dimensional multivariate normal distribution with a mean vector *µ* and a correlation matrix Σ. We consider, in particular, three structures of the correlation matrix: blockwise positive correlation, autoregressive (AR) type correlation and blockwise mixed correlation.

### Blockwise correlation

Let the columns of the data matrix *Y*_(*N* X M)_, be divided into *B* blocks, where each block contains *M*/*B* metabolites. The partitioned correlation matrix has the form

$$ {\displaystyle \sum } = \left(\begin{array}{ccc}\hfill {\Sigma}_{11}\hfill & \hfill \dots \hfill & \hfill {\Sigma}_{1 B}\hfill \\ {}\hfill \vdots \hfill & \hfill \ddots \hfill & \hfill \vdots \hfill \\ {}\hfill {\Sigma}_{B1}\hfill & \hfill \dots \hfill & \hfill {\Sigma}_{B B}\hfill \end{array}\right) $$

The matrices Σ_*ii*_ are determined by the pairwise correlations *ρ*_*w*_, such that all the components have a within block correlation of *ρ*_*w*_. The matrices Σ_*ij*_, *i* ≠ *j*, are determined by the pairwise correlations *ρ*_off_; that is, all the components have a between block correlation *ρ*_off_. The two types of blockwise correlation matrices used in this study are one with all positive correlations where the *ρ*_*w*_ is positive only and the other is mixed where Σ_*ii*_ contains both positive and negative correlations. The mixed correlation has the form which is blockwise split in half where the diagonal blocks are positively correlated and the off-diagonal blocks are negatively correlated. For example, if Σ_*ii*_ contained six metabolites for any *i*, the matrix Σ_*ii*_ would be:

$$ {\displaystyle \sum_{ii}} = \left(\begin{array}{ccc}\hfill \begin{array}{cc}\hfill 1\hfill & \hfill +\hfill \\ {}\hfill +\hfill & \hfill 1\hfill \\ {}\hfill +\hfill & \hfill +\hfill \end{array}\hfill & \hfill \begin{array}{cc}\hfill +\hfill & \hfill -\hfill \\ {}\hfill +\hfill & \hfill -\hfill \\ {}\hfill 1\hfill & \hfill -\hfill \end{array}\hfill & \hfill \begin{array}{cc}\hfill -\hfill & \hfill -\hfill \\ {}\hfill -\hfill & \hfill -\hfill \\ {}\hfill -\hfill & \hfill -\hfill \end{array}\hfill \\ {}\hfill \begin{array}{cc}\hfill -\hfill & \hfill -\hfill \\ {}\hfill -\hfill & \hfill -\hfill \\ {}\hfill -\hfill & \hfill -\hfill \end{array}\hfill & \hfill \begin{array}{cc}\hfill -\hfill & \hfill 1\hfill \\ {}\hfill -\hfill & \hfill +\hfill \\ {}\hfill -\hfill & \hfill +\hfill \end{array}\hfill & \hfill \begin{array}{cc}\hfill +\hfill & \hfill +\hfill \\ {}\hfill 1\hfill & \hfill +\hfill \\ {}\hfill +\hfill & \hfill 1\hfill \end{array}\hfill \end{array}\right) $$

where the + is the positive *ρ*_*w*_ and − is the negative *ρ*_*w*_

### Autoregressive-type correlation

The other correlation structure used is the autoregressive type correlation. An AR correlation matrix of order one is defined by pairwise correlations *ρ* ^|*i* − *j*|^, for metabolites *i*, *j* = 1, …, *M*.

The combinations used were (*N* [Samples] X *M* [Metabolites]) = 20 X 400, 50 X 400, and 100 X 900. The means of the metabolites are assumed to be different and are generated from a Uniform(−5, 5) distribution. The metabolites within each block were strongly correlated with *ρ*_*w*_ = 0.7, but nearly uncorrelated with metabolites in other blocks, *ρ*_off_ = 0.2. In the AR type correlation *ρ* = 0.9. For the degree of missing, three levels were studied: 9% missing, 15% missing and 30% missing. Missing data were created based on the two kinds of missingness, MNAR and MAR (technically the latter are generated by MCAR, though a MAR mechanism can be exploited for imputation since the metabolite values are highly correlated). Within each level of missing, a one-third and two-third combination was used to create both MNAR and MAR. We looked at the scenario where MNAR is greater than MAR and vice versa. For example in 9% missing, we considered 6% as MNAR and 3% as MAR and then considered 6% as MAR and 3% as MNAR. Data below the given MNAR percent was considered as missing and the MAR percent was randomly generated in the non-missing data. The datasets with missing values were passed through a cleaning process where metabolites with more than 75% missing observations were eliminated individually. Throughout, the number of neighbors *K* used for imputation was set to 10. We evaluated three *K*’s (*K* = 5, 10 and 20) and found consistency in *K* = 10 as it gave the best RMSE values.

#### Real data studies

##### Myocardial infarction data

We use the in vivo metabolomics data on myocardial infarction (MI). The data consists of two groups, MI vs control, five samples in each group and 288 metabolites. Adult mice were subjected to permanent coronary occlusion (myocardial infarction; MI) or Sham surgery. Adult C57BL/6 J mice from The Jackson Laboratory (Bar Harbor, ME) were used in this study and were anesthetized with ketamine (50 mg/kg, intra-peritoneal) and pentobarbital (50 mg/kg, intra-peritoneal), orally intubated with polyethylene-60 tubing, and ventilated (Harvard Apparatus Rodent Ventilator, model 845) with oxygen supplementation prior to the myocardial infarction. The study was aimed to examine the metabolic changes that occur in the heart in vivo during heart failure using mouse models of permanent coronary ligation. A combination of liquid chromatography (LC) MS/MS and gas chromatography (GC) MS techniques was used to measure the 288 metabolites in these hearts. The MS was based on a Waters ACQUITY UPLC and a Thermo-Finnigan LTQ mass spectrometer, which consisted of an electrospray ionization source and linear ion trap mass analyzer. The cases had 220 metabolites with complete values, six metabolites with complete missing and 62 metabolites had 4.8% missing values whereas the controls had 241 metabolites with complete values, seven metabolites with complete missing and 40 metabolites had 7.8% missing values. The LOD for this dataset is considered as the minimum value of the dataset as commonly used in untargeted metabolomics. Details of the experiments are described in Sansbury et al. [15].

##### Atherothrombotic data

We use the human atherothrombotic myocardial infarction (MI) metabolomics data. The data was identified between two groups, those with acute MI and those with stable coronary artery disease (CAD). Acute MI was further stratified into thrombotic (Type1) and non-thrombotic (Type2) MI. The data was collected across four time points and for the context of this research we used the baseline data only. The three groups, sCAD, Type1 and Type2 had 15, 11, and 12 patients with 1032 metabolites. The sCAD had 685 metabolites with complete values, 39 metabolites with complete missing, and 308 metabolites had 10.2% missing, the Type1 group had 689 metabolites with complete values, 43 metabolites with complete missing and 300 metabolites had 9.8% missing whereas the Type2 group had 610 metabolites with complete values, 66 metabolites with complete missing and 356 metabolites had 12.3% missing. The LOD for this dataset is considered as the minimum value of the dataset as commonly used in untargeted metabolomics. Plasma samples collected from the patients were used and 1032 metabolites were detected and quantified by GC-MS and ultra-performance (UP) LC-MS in both positive and negative ionization modes. Details of the experiment are described in DeFilippis et al. [23].

##### African race data

We used the African Studies data which is publicly available on The Metabolomics WorkBench. This data is available at the NIH Common Fund’s Data Repository and Coordinating Center (supported by NIH grant, U01-DK097430) website (http://www.metabolomicsworkbench.org), where it has been assigned a Metabolomics Workbench Project ID: PR000010. The data is directly accessible from The Metabolomics WorkBench database [16]. The data was collected to compare metabolomics, phenotypic and genetic diversity across various groups of Africans. The data consisted of 40 samples; 25 samples from Ethiopia and 15 samples from Tanzania and 5126 metabolites. For the purpose of this study we made sure we had a complete dataset in order to compare the methods. The complete datasets created were two datasets based on the country; Ethiopia dataset (25 samples by 1251 metabolites) and Tanzania dataset (15 samples by 2250 metabolites).

Due to small sample sizes in metabolomics datasets, we used a simulation approach originally designed to resemble the multivariate distribution of gene expression in the original microarray data [24]. Since our Myocardial Infarction and Atherothrombotic data had missing values we first imputed missing values based on the KNN-CR method and then used the simulation method to simulate 100 datasets. For the African Race data we started with a complete dataset. The different groups were considered as independent datasets and the imputation was done on them separately. We used the similar mechanism for missingness and screening as used in the simulation studies, with sample sizes of 25 and 50 for the myocardial infarction dataset, 50 and 100 for the human atherothrombotic dataset and 15 and 25 for the Tanzania and Ethiopia data sets, respectively, from the African race study.

## Results

### Simulation results

**Fig. 3:**
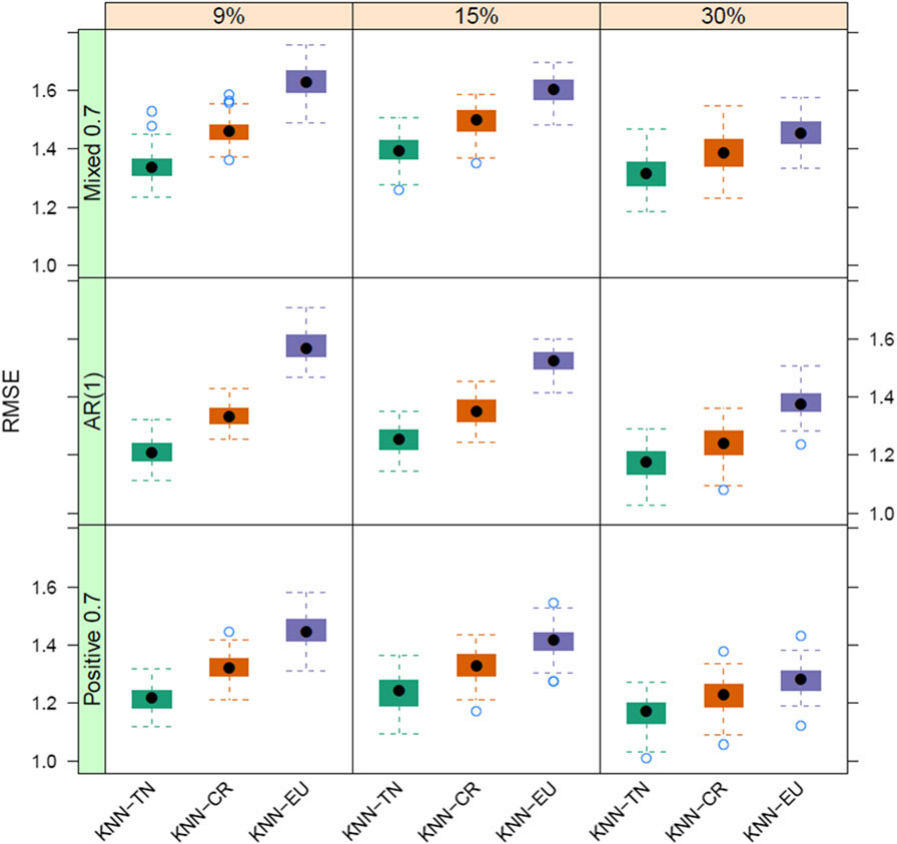
Boxplots of root mean squared error for KNN-TN, KNN-CR and KNN-EU for 100 datasets, 20 samples by 400 metabolites. Total missing was considered at 9%, 15% and 30% and within each missing MNAR is greater than MAR. The three correlation structure used was i) only positive correlation 0.7, ii) AR(1) correlation 0.9 and iii) mixed correlation 0.7

**Fig. 4:**
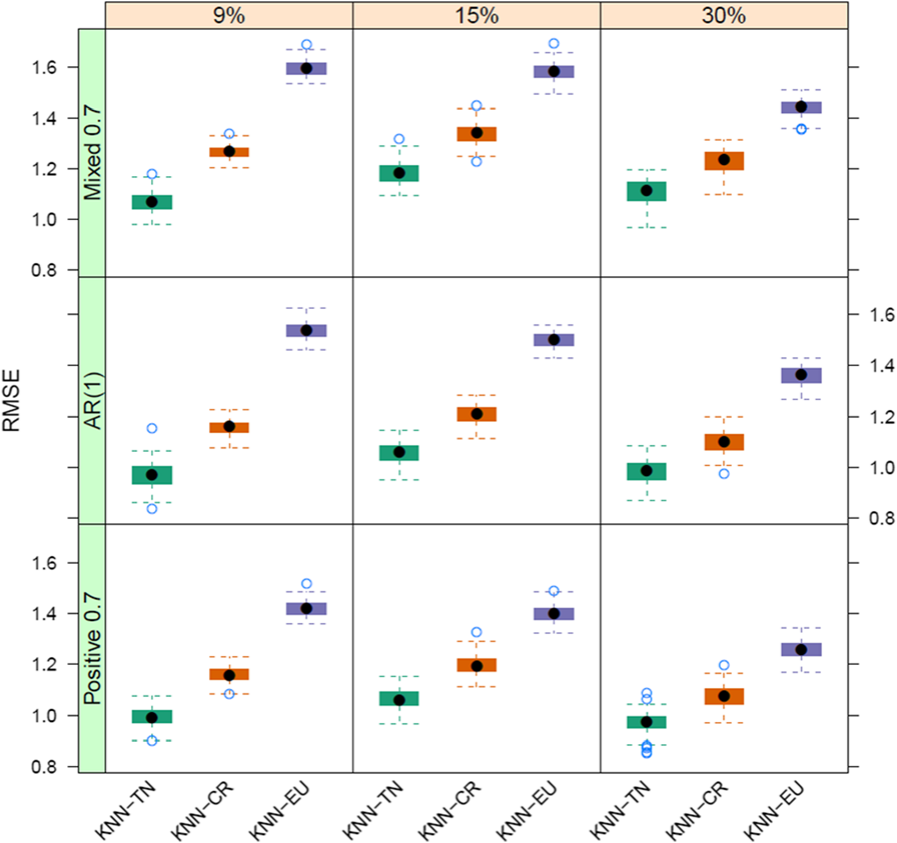
Boxplots of root mean squared error for KNN-TN, KNN-CR and KNN-EU for 100 datasets, 50 samples by 400 metabolites. Total missing was considered at 9%, 15% and 30% and within each missing MNAR is greater than MAR. The three correlation structure used was i) only positive correlation 0.7, ii) AR(1) correlation 0.9 and iii) mixed correlation 0.7

**Fig. 5:**
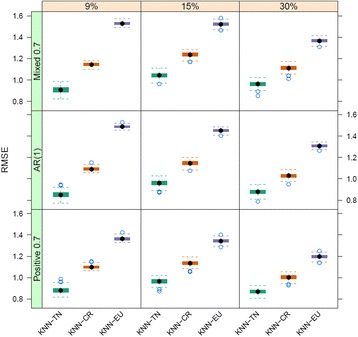
Boxplots of root mean squared error for KNN-TN, KNN-CR and KNN-EU for 100 datasets, 100 samples by 900 metabolites. Total missing was considered at 9%, 15% and 30% and within each missing MNAR is greater than MAR. The three correlation structure used was i) only positive correlation 0.7, ii) AR(1) correlation 0.9 and iii) mixed correlation 0.7

**Fig. 6:**
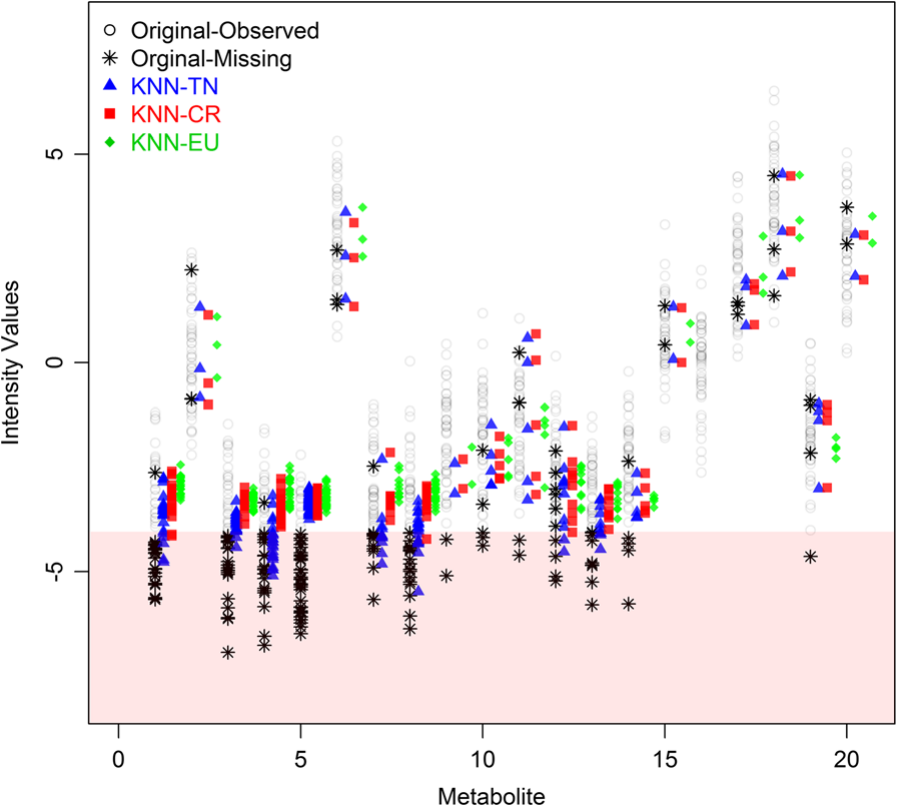
Comparison of the true missing values with missing values imputed from the three methods based on a single simulated dataset (*N* = 50 X M = 400). The values for the first 20 metabolites are shown. The x-axis represents the metabolites, and the y-axis represents the intensity values. The *open black circles* represent observed values, while the *black stars* represent missing observations. *Blue triangles*, *red squares*, and *green diamonds* represent missing values imputed by KNN-TN, KNN-CR and KNN-EU, respectively. The region below the LOD is shaded in *light red*. In most cases, the KNN-TN algorithm is able to impute missing values below the LOD better than the other two methods (e.g., metabolites 1, 3, 4, 7, 8, 12, and 13). In other cases, the KNN-TN imputations are similar to KNN-CR (e.g., for metabolite 5, for which the missing below the LOD was too high and the NR algorithm was unable to converge)

In this section, we present the results of the simulation studies comparing the performance measures of KNN-TN, KNN-CR and KNN-EU. Figures 3, 4 and 5 plot the distribution of the RMSE values for KNN-TN, KNN-CR and KNN-EU by correlation type and percent missing for sample sizes 20, 50, and 100, respectively. Since the pattern of results was similar regardless of whether the percent MNAR was less than the percent MAR, results are shown for percent MNAR > percent MAR only. As can be seen from the figures, the results consistently show that the KNN-TN method outperforms both the KNN-CR and KNN-EU methods. ANOVA modeling of the RMSE values shows statistically significant differences between the KNN-TN method and KNN-CR/KNN-EU methods for all three cases, and significant effects for the other two factors (percent missing and correlation type) as well (Additional file 2: Tables S1–S3). To visualize how our method works we selected a simulated dataset from *N* = 50 and M = 400 with 15% missing (10% below the LOD and 5% MAR) and compared the true missing values with KNN-TN, KNN-CR and KNN-EU. Figure 6 demonstrates that our imputation method imputes values below the limit of detection whereas the Euclidean or correlation based metrics are less accurate for these values. The figure is reproducible with our included example script in Additional file 3. We further compared the three methods with standard imputation methods in metabolomics (zero, minimum and mean imputation methods) and all three KNN imputation algorithms outperformed the standard methods. The results for the simulation studies are shown in Additional file 2: Tables S4–S6 where we see the average RMSE range was from 4.0 to 5.8.

### Real data simulations results

We conducted a simulation study based on the real datasets to further validate our results. Tables 1, 2, and 3 show the results of the in vivo myocardial infarction data, human atherothrombotic data, and publicly available African Race data. In all cases the KNN-TN and KNN-CR results are substantially better than the KNN-EU results, with RMSE means more than two standard deviations below the means for KNN-EU (*p*-value < 0.05 for KNN-TN vs. KNN-EU contrast, Additional file 2: Tables S7–S9). The difference between KNN-TN and KNN-CR is much smaller by comparison, with statistically significant differences only for the Atherothrombotic and African Race data sets. However, in every case the mean RMSE for KNN-TN is below that for KNN-CR. Additional file 2: Tables S7–S9 show that significant differences in RMSE values exist according to the other factors in the simulation study (percent missing, group, and sample size) as well. We further compared the three methods the standard imputation methods in metabolomics (zero, minimum and mean imputation methods) and all three KNN imputation algorithms outperformed the standard methods. The results for the real data are shown in Additional file 2: Tables S10–S12 where we see the average RMSE range was from 2.2 to 7.2. The *t*-test analysis and the MLCI values are shown in Table 4. A higher value of MLCI indicates that the metabolite list from the imputed data is similar to that from the complete data and from the tables KNN-TN and KNN-CR have the highest values, whereas the KNN-EU, Zero, Minimum and Mean imputation methods have lower MLCI indexes. Differences in mean MLCI values between KNN-TN and KNN-CR were not statistically significant (Additional file 2: Tables S13–S15), whereas KNN-TN was significantly better than the other four methods in all cases except for the African Race data (where mean imputation and all KNN imputation methods were roughly equivalent and better than zero and minimum value imputation).

**Table 1:** Average RMSE of 100 simulations using the in vivo myocardial infarction dataset for KNN-TN, KNN-CR and KNN-EU

MNAR/MAR	Sample size	Group	KNN-TN	KNN-CR	KNN-EU
6%/3%	25	Cases	0.613 (0.072)	0.619 (0.071)	0.786 (0.075)
	25	Controls	0.436 (0.054)	0.441 (0.054)	0.607 (0.047)
	50	Cases	0.597 (0.045)	0.602 (0.046)	0.776 (0.048)
	50	Controls	0.415 (0.032)	0.420 (0.031)	0.600 (0.028)
10%/5%	25	Cases	0.632 (0.099)	0.637 (0.101)	0.810 (0.087)
	25	Controls	0.416 (0.052)	0.419 (0.050)	0.555 (0.044)
	50	Cases	0.607 (0.073)	0.610 (0.073)	0.809 (0.069)
	50	Controls	0.409 (0.034)	0.412 (0.034)	0.556 (0.029)
20%/10%	25	Cases	0.610 (0.108)	0.612 (0.107)	0..701 (0.091)
	25	Controls	0.381 (0.059)	0.389 (0.058)	0.498 (0.048)
	50	Cases	0.586 (0.083)	0.586 (0.081)	0.699 (0.071)
	50	Controls	0.370 (0.053)	0.381 (0.053)	0.499 (0.041)

Total missing was considered at 9%, 15% and 30%, and within each missing, MNAR was greater than MAR

**Table 2:** Average RMSE of 100 simulations using the human atherothrombotic dataset for KNN-TN, KNN-CR and KNN-EU

MNAR/MAR	Sample size	Group	KNN-TN	KNN-CR	KNN-EU
6%/3%	50	sCAD	1.145 (0.047)	1.171 (0.046)	1.410 (0.052)
	50	TYPE1	1.255 (0.054)	1.273 (0.053)	1.555 (0.057)
	50	TYPE2	1.266 (0.051)	1.279 (0.050)	1.567 (0.055)
	100	sCAD	1.083 (0.048)	1.109 (0.041)	1.403 (0.053)
	100	TYPE1	1.183 (0.048)	1.199 (0.041)	1.531 (0.053)
	100	TYPE2	1.183 (0.048)	1.191 (0.041)	1.531 (0.053)
10%/5%	50	sCAD	1.146 (0.045)	1.168 (0.045)	1.337 (0.050)
	50	TYPE1	1.262 (0.059)	1.280 (0.057)	1.490 (0.059)
	50	TYPE2	1.296 (0.048)	1.315 (0.047)	1.531 (0.051)
	100	sCAD	1.075 (0.031)	1.095 (0.031)	1.330 (0.034)
	100	TYPE1	1.171 (0.039)	1.189 (0.038)	1.460 (0.041)
	100	TYPE2	1.189 (0.040)	1.207 (0.038)	1.490 (0.040)
20%/10%	50	sCAD	1.120 (0.049)	1.140 (0.049)	1.210 (0.047)
	50	TYPE1	1.261 (0.061)	1.282 (0.061)	1.398 (0.059)
	50	TYPE2	1.354 (0.058)	1.373 (0.058)	1.484 (0.054)
	100	sCAD	1.033 (0.035)	1.053 (0.035)	1.198 (0.034)
	100	TYPE1	1.153 (0.041)	1.176 (0.041)	1.372 (0.041)
	100	TYPE2	1.246 (0.037)	1.266 (0.037)	1.451 (0.036)

Total missing was considered at 9%, 15% and 30%, and within each missing, MNAR was greater than MAR

**Table 3:** Average RMSE of 100 simulations using the African Race dataset for KNN-TN, KNN-CR and KNN-EU

MNAR/MAR	Sample size	Group	KNN-TN	KNN-CR	KNN-EU
6%/3%	15	Tanzania	0.695 (0.050)	0.711 (0.051)	0.772 (0.049)
	25	Ethiopia	0.575 (0.029)	0.592 (0.029)	0.701 (0.033)
10%/5%	15	Tanzania	0.659 (0.052)	0.674 (0.053)	0.728 (0.050)
	25	Ethiopia	0.556 (0.029)	0.574 (0.029)	0.665 (0.031)
20%/10%	15	Tanzania	0.577 (0.049)	0.588 (0.049)	0..627 (0.051)
	25	Ethiopia	0.507 (0.026)	0.520 (0.027)	0.599 (0.028)

Total missing was considered at 9%, 15% and 30%, and within each missing, MNAR was greater than MAR

**Table 4:** Average MLCI of 100 simulations using the Myocardial, Atherothrombotic and African Race dataset with 15% missingness

Imputation Method	Myocardial Dataset	Atherothrombotic Dataset	African Race Dataset
	MLCI	MLCI	MLCI
Zero	0.061 (0.054)	0.086 (0.026)	0.028 (0.026)
Min	0.396 (0.061)	0.248 (0.069)	0.135 (0.078)
Mean	0.440 (0.089)	0.368 (0.103)	0.266 (0.134)
KNN-TR	0.510 (0.097)	0.392 (0.110)	0.274 (0.140)
KNN-CR	0.504 (0.094)	0.391 (0.110)	0.274 (0.139)
KNN-EU	0.474 (0.091)	0.380 (0.110)	0.272 (0.139)

The Myocardial dataset was comparing cases and controls of sample size 25 in each group, Atherothrombotic dataset was comparing sCAD and Type 2 MI of sample size 50 in each group and the African Race dataset was comparing Tanzania and Ethiopia of sample size 15 and 25 respectively

## Discussion

The objective of this study was to develop an approach for imputing missing values in data generated by mass spectrometry. When metabolites occur at low abundance, below the detection limit of the instrumentation, we can consider it as missing not at random. In contrast, missing values resulting from technical errors are considered missing at random. To this end, we introduce an extension to the KNN imputation algorithm which handles truncated data, termed KNN-TN. To our knowledge, this is the first paper to propose a hybrid KNN imputation approach which can simultaneously handle missing data generated by both MNAR (falling below the LOD) and MAR mechanisms. Since MNAR is involved and is due to the detection limit, we consider the detection limit as a truncation point and assume that the metabolite follows a truncated normal distribution. Therefore the mean and standard deviation are estimated from the truncated normal distribution and used to standardize the metabolites in the KNN imputation algorithm. The simulation results show that the proposed method performs better than KNN based on correlation or Euclidean measures when there is missing data due to a threshold LOD.

In our simulations we evaluated three different data set sizes: small (20 samples by 400 metabolites), medium (50 samples by 400 metabolites) and large (100 samples by 900 metabolites). As the sample size increased, the RMSE was lower for the different missing percentages. The LOD was calculated based on the missing percentage. For instance in 9% missing (where 6% was considered as MNAR) the 6% quantile for the complete data was considered as the LOD where we considered everything below that value as missing. For the simulation studies, the results shown in the tables are based on when the MNAR percentage is greater than the MAR percentage (e.g., for 9% total missing, 6% is MNAR and 3% is MAR). However the results were similar when the MAR percentage was greater than the MNAR percentage, with KNN-TN outperforming both KNN-CR and KNN-EU. In our results, when MNAR is greater than MAR we typically observed the RMSE was greatest at 15% MVs whereas it was lowest at 30% MVs. This counter-intuitive result is likely due to the fact that in the cleaning process (which removes metabolites with >75% MVs) we are removing more metabolites whose values are concentrated near the LOD. For example in the case of 50 samples by 400 metabolites, after screening we reduced the metabolites to an average of about 387 metabolites for 15% missing and 345 metabolites for 30% missing. When the MAR was greater than MNAR, the RMSE increased with the increase in MV percentage.

Troyanskaya et al. [9] evaluated a number of different missing value imputation methods and suggested the KNN method to be more robust and sensitive compared to the other methods. In another study by Brock et al. [12], they compared the KNN based on two different neighbor selection metrics, Euclidean and Correlation and concluded that the correlation based neighbor selection performed better than the Euclidean neighbor selection in most of the cases. In this study we focused on enhancing the KNN method specifically for imputing values when there is missing due to an LOD. Future studies will evaluate how these methods compare to other imputation algorithms in this setting.

Recently, several studies have investigated imputation for MS data [13, 14, 25]. Taylor et al. [25] evaluated seven different imputation methods (half minimum, mean, KNN, local least squares regression, Bayesian principal components analysis, singular value decomposition and random forest) and its effects on multiple biological matrix analyses, more specifically on the within-subject correlation of compounds between biological matrices and its consequences on MANOVA results. They concluded that no imputation method was superior but the mean and half minimum performed poorly. Gromski et al. [13] looked at five different imputation methods (zero, mean, median, KNN and random forest) and its influence on unsupervised and supervised learning. Their results recommended that random forest is better than the other imputation methods and it provided better results in terms of classification rates for both principal components-linear discriminant analysis and partial least squares-discriminant analysis. Hrydziuszko et al. [14] suggested the need of missing value imputation as an important step in the processing pipeline. They used metabolomics datasets based on infusion Fourier transform ion cyclotron resonance mass spectrometry and compared eight different imputation methods (predefined value, half minimum, mean, median, KNN, Bayesian Principal Component Analysis, Multivariate Imputation, and REP). Based on their findings, KNN performed better than the other methods.

We included a preliminary investigation of the impact of MV imputation on downstream statistical analysis of metabolomics data. While the KNN-TN method was significantly better than four other imputation algorithms (zero imputation, minimum value imputation, and KNN-EU imputation) in two of three data sets, it was no better than KNN-EU imputation. Further, on the African Race data set there was no significant difference between any of the KNN imputation algorithms and mean imputation, though all were better than zero and minimum value imputation. Although this result is somewhat disappointing, a more comprehensive study of all potential downstream analyses is needed to fully determine, whether the improved imputation accuracy of the KNN-TN method translates into better downstream statistical analysis, and the characteristics of data sets for which more advanced imputation algorithms offer a decided advantage [22].

In some cases (high percent missing or small sample size) the variability of the RMSE for KNN-TN is higher than or similar to that for KNN-CR. This is directly related to the estimation of the mean and variance for the truncated normal distribution, which can be difficult when there are excessive amounts of missing data. In fact, for sample sizes less than 20 there is little to no gain in using KNN-TN over KNN-CR, unless the missing percentage is below the values evaluated in this study (data not shown). To stabilize the estimation of these parameters, one possibility is to again borrow information from metabolites having similar intensity profiles. This is akin to the empirical Bayes approach used to fit linear models and generalized linear models in microarray and RNA-seq studies [26–29]. Our future research will explore this possibility for improving the KNN-TN algorithm.

A related limitation is the reliance on the normality assumption for estimating the truncated mean and standard deviation. In our simulation study we investigated data from a normal distribution, whereas in many cases metabolite data will be non-normally distributed. In these cases we suggest to first transform the data to normality, then impute the values and lastly transform back. As seen in our real datasets, the metabolites are not normally distributed and we log transform them to approximately achieve normality prior to imputation.

The likelihood used in our KNN-TN method is based solely on the observed metabolite data. The full data likelihood would include missing data as well. This is difficult to specify in the current situation as the mechanism by which the MVs were generated (e.g., MNAR, MAR, or MCAR) is unknown. It is possible to improve the algorithm by incorporating these MVs directly into the likelihood function, but ancillary information (e.g., from metabolites determined to be neighbors) is necessary to inform the system regarding the missingness mechanism (e.g., via the EM-algorithm).

## Conclusion

In conclusion, the experimental results reveal that compared with KNN based on correlation and Euclidean metrics, KNN based on truncation estimation is a competitive approach for imputing high dimensional data where there is potential missingness due to a truncation (detection) threshold. Results based on both real and simulated experimental data show that the proposed method (KNN-TN) generally has lower RMSE values compared to the other two KNN methods and simpler imputation algorithms (zero, mean, and minimum value imputation) when there is both missing at random and missing due to a threshold value. Assessment based on concordance in statistical significance testing demonstrate that KNN-TN and KNN-CR are roughly equivalent and generally outperform the other four methods. However, the approach has limitations with smaller sample sizes, unless the missing percentage is also small. Lastly, even though this study is based on metabolomic datasets our findings are more generally applicable to high-dimensional data that contains missing values associated with an LOD, for instance proteomics data and delta-CT values from qRT-PCR array cards [30].

## Additional files

Additional file 1: Details of the Newton Raphson procedure for estimating the mean and standard deviation of a truncated normal distribution. (DOCX 21 kb)
Additional file 2: Tables S1. through **Table S15.** including results for ANOVA testing of differences in RMSE values, results for the zero, mean and minimum imputation methods and results for ANOVA testing of differences in MCLI values. (DOC 244 kb)
Additional file 3: R script illustrating providing a working example. (R 4 kb)
Additional file 4: R code for the kNN imputation functions. (R 13 kb)
Additional file 5: R code for the Simulation datasets. (R 2 kb)

## Abbreviations

AR: Autoregressive
GC: Gas chromatography
iid: Independent and identically distributed
KNN: K-nearest neighbor
KNN-CR: K-nearest neighbor correlation
KNN-EU: K-nearest neighbor Euclidean
KNN-TN: K-nearest neighbor truncation
LC: Liquid chromatography
LOD: Detection limit
MAR: Missing at random
MCAR: Missing completely at random
MI: Myocardial infarction
MLCI: Metabolite list concordance index
MLE: Maximum likelihood estimators
MNAR: Missing not at random
MS: Mass spectrometry
MV: Missing values
NR: Newton Raphson
RMSE: Root mean square error
S/N: Signal to noise ratio
sCAD: Stable coronary artery disease
SD: Standard deviation
UP: Ultra-performance
