# Supplementary material for: Distribution based nearest neighbor imputation for truncated high dimensional data with applications to pre-clinical and clinical metabolomics studies
Source: BMC Bioinformatics. 2017 Feb 20;18:114. doi: 10.1186/s12859-017-1547-6 (PMC5319174; doi:10.1186/s12859-017-1547-6)
Supplement: Additional file 1: — Details of the Newton Raphson procedure for estimating the mean and standard deviation of a truncated normal distribution. (DOCX 21 kb) [file 12859_2017_1547_MOESM1_ESM.docx]

**Additional file 1**

**Details of NR Procedure**

For notational convenience we define the probability $P\left( Y\in\left( a,\infty\right) | \mu,\sigma^{2} \right)$ as

$$\zeta\left( \mu,\sigma^{2} \right)= \int_{a}^{\infty} \frac{1}{\sqrt{2\pi\sigma^{2}}}e^{\frac{-{(y-\mu)}^{2}}{2\sigma^{2}}}dy$$

Interchanging differentiation and integration, the first derivatives of the above equation with respect to $\mu$ and $\sigma$ are

$${\zeta'}_{\mu}= \int_{a}^{\infty} e^{\frac{-{(y-\mu)}^{2}}{2\sigma^{2}}}\times\frac{(y-\mu)}{\sigma^{3}\sqrt{2\pi}} dy, \mathrm{and}$$

$${\zeta'}_{\sigma}= \int_{a}^{\infty} e^{\frac{-{(y-\mu)}^{2}}{2\sigma^{2}}}\times\left( \frac{{(y-\mu)}^{2}}{\sigma^{4}\sqrt{2\pi}}-\frac{1}{\sigma^{2}\sqrt{2\pi}} \right) dy$$

Using the above derivatives, the gradient (G) (first partial derivative) with respect to the parameters is

$$\boldsymbol{G}= \left[ \begin{matrix} \frac{\partial l}{\partial\mu} \\ \frac{\partial l}{\partial\sigma} \end{matrix} \right]= \left[ \begin{matrix} g_{1} \\ g_{2} \end{matrix} \right]=\left[ \begin{matrix} -n\frac{{\zeta^{'}}_{\mu}}{\zeta}-\frac{1}{\sigma^{2}}(n\mu-\sum y_{i}) \\ -n\frac{{\zeta'}_{\sigma}}{\zeta}-\frac{n}{\sigma}+\frac{\sum{(y_{i}-\mu)}^{2}}{\sigma^{3}} \end{matrix} \right]$$

The second derivatives for the Hessian are

$${\zeta''}_{\mu}\left( \mu,\sigma^{2} \right)=\frac{\partial^{2}\zeta}{\partial^{2}\mu}= \int_{a}^{\infty} e^{\frac{-{(y-\mu)}^{2}}{2\sigma^{2}}}\times\left( \frac{{(y-\mu)}^{2}}{\sigma^{5}\sqrt{2\pi}}-\frac{1}{\sigma^{3}\sqrt{2\pi}} \right)dy,$$

$${\zeta^{''}}_{\sigma}\left( \mu,\sigma^{2} \right)=\frac{\partial^{2}\zeta}{\partial^{2}\sigma}= \int_{a}^{\infty} e^{\frac{-\left( y-\mu\right)^{2}}{2\sigma^{2}}}\times\left( \frac{\left( y-\mu\right)^{2}}{\sigma^{7}\sqrt{2\pi}}-\frac{5\left( y-\mu\right)^{2}}{\sigma^{5}\sqrt{2\pi}}+\frac{2}{\sigma^{3}\sqrt{2\pi}} \right)dy, \mathrm{and}$$

$${\psi^{''}}_{\mu,\sigma}\left( \mu,\sigma^{2} \right)=\frac{\partial^{2}\psi}{\partial\mu\partial\sigma}= \int_{a}^{\infty} e^{\frac{-\left( y-\mu\right)^{2}}{2\sigma^{2}}}\times\left( \frac{\left( y-\mu\right)^{3}}{\sigma^{6}\sqrt{2\pi}}-\frac{3(y-\mu)}{\sigma^{4}\sqrt{2\pi}} \right)dy$$

$${\zeta^{''}}_{\mu,\sigma}\left( \mu,\sigma^{2} \right)=\frac{\partial^{2}\zeta}{\partial\mu\partial\sigma}= \int_{a}^{\infty} e^{\frac{-\left( y-\mu\right)^{2}}{2\sigma^{2}}}\times\left( \frac{\left( y-\mu\right)^{3}}{\sigma^{6}\sqrt{2\pi}}-\frac{3(y-\mu)}{\sigma^{4}\sqrt{2\pi}} \right)dy$$

Using the equations above and taking the derivatives, the Hessian matrix is

$$\boldsymbol{H}= \left[ \begin{matrix} \frac{\partial g_{1}}{\partial\mu} & \frac{\partial g_{1}}{\partial\sigma} \\ \frac{\partial g_{2}}{\partial\mu} & \frac{\partial g_{2}}{\partial\sigma} \end{matrix} \right]= \begin{matrix} -n\frac{{\zeta\zeta''}_{\mu}-{({\zeta^{'}}_{\mu})}^{2}}{\zeta^{2}}-\frac{n}{\sigma^{2}} & -n\frac{{\zeta\zeta^{''}}_{\sigma|\mu}-{\zeta^{'}}_{\mu}{\zeta^{'}}_{\sigma}}{\zeta^{2}}+\frac{2(n\mu-\sum y_{i})}{\sigma^{3}} \\ -n\frac{{\zeta\zeta''}_{\mu|\sigma}-{\zeta^{'}}_{\mu}{\zeta^{'}}_{\sigma}}{\zeta^{2}}+\frac{2(n\mu-\sum y_{i})}{\sigma^{3}} & -n\frac{{{\zeta\zeta}^{''}}_{\sigma}-\left( {\zeta^{'}}_{\sigma} \right)^{2}}{\zeta^{2}}+\frac{n}{\sigma^{2}}-\frac{3(n\mu-\sum y_{i})}{\sigma^{4}} \end{matrix}$$

The Newton Raphson updating equation is then

$${\left( \mu,\sigma^{2} \right)^{T}}_{n+1}= {\left( \mu,\sigma^{2} \right)^{T}}_{n}-\boldsymbol{H}_{n}^{-1}\boldsymbol{G}_{n} ,$$

where ${\left( \mu,\sigma^{2} \right)^{T}}_{n}$, $\boldsymbol{H}_{n}$, and $\boldsymbol{G}_{n}$ are the estimates, Hessian matrix, and gradient, respectively, at step $n$ of the procedure and ${\left( \mu,\sigma^{2} \right)^{T}}_{n+1}$ is the estimate at step $n+1$.
