# Supplementary material for: Distribution based nearest neighbor imputation for truncated high dimensional data with applications to pre-clinical and clinical metabolomics studies
Source: BMC Bioinformatics. 2017 Feb 20;18:114. doi: 10.1186/s12859-017-1547-6 (PMC5319174; doi:10.1186/s12859-017-1547-6)
Supplement: Additional file 2: Tables S1. — through Table S15. including results for ANOVA testing of differences in RMSE values, results for the zero, mean and minimum imputation methods and results for ANOVA testing of differences in MCLI values. (DOC 244 kb) [file 12859_2017_1547_MOESM2_ESM.doc]

**Additional file 2**

**Table S1**

**a.** Specific differences in RMSE values for the KNN-CR and KNN-EU methods compared to the KNN-TR method for 20 samples by 400 metabolites.

| **Contrast** | **Estimate** | **Std. Error** | **t Value** | **P Value** |
| --- | --- | --- | --- | --- |
| **KNN-CR – KNN-TN** | 0.094 | 0.002815 | 33.303 | <2e-16 *** |
| **KNN-EU – KNN-TN** | 0.224 | 0.002815 | 79.519 | <2e-16 *** |

**b. ANOVA table giving the significance of the three factors in the simulation study for 20 samples by 400 metabolites.**

|  | **Df** | **Sum Sq** | **Mean Sq** | **F value** | **P Value** |
| --- | --- | --- | --- | --- | --- |
| **Imputation Method** | 2 | 22.752 | 11.376 | 3189.4 | <2e-16 *** |
| **Percent Missing** | 2 | 6.574 | 3.287 | 921.6 | <2e-16 *** |
| **Correlation Type** | 2 | 12.333 | 6.166 | 1728.8 | <2e-16 *** |
| **Residuals** | 2693 | 9.605 | 0.004 |  |  |

**Table S2**

**a.** Specific differences in RMSE values for the KNN-CR and KNN-EU methods compared to the KNN-TR method for 50 samples by 400 metabolites.

|  | **Estimate** | **Std. Error** | **t Value** | **P Value** |
| --- | --- | --- | --- | --- |
| **KNN-CR – KNN-TN** | 0.148 | 0.002517 | 58.72 | <2e-16 *** |
| **KNN-EU – KNN-TN** | 0.224 | 0.002517 | 163.23 | <2e-16 *** |

**b. ANOVA table giving the significance of the three factors in the simulation study for 50 samples by 400 metabolites.**

|  | **Df** | **Sum Sq** | **Mean Sq** | **F value** | **P Value** |
| --- | --- | --- | --- | --- | --- |
| **Imputation Method** | 2 | 77.95 | 38.98 | 13672 | <2e-16 *** |
| **Percent Missing** | 2 | 5.83 | 2.92 | 1023 | <2e-16 *** |
| **Correlation Type** | 2 | 9.71 | 4.85 | 1703 | <2e-16 *** |
| **Residuals** | 2693 | 7.68 | 0.00 |  |  |

**Table S3**

**a.** Specific differences in RMSE values for the KNN-CR and KNN-EU methods compared to the KNN-TR method for 100 samples by 900 metabolites.

|  | **Estimate** | **Std. Error** | **t Value** | **P Value** |
| --- | --- | --- | --- | --- |
| **KNN-CR – KNN-TN** | 0.186 | 0.002278 | 81.60 | <2e-16 *** |
| **KNN-EU – KNN-TN** | 0.473 | 0.002278 | 207.60 | <2e-16 *** |

**b. ANOVA table giving the significance of the three factors in the simulation study for 100 samples by 900 metabolites.**

|  | **Df** | **Sum Sq** | **Mean Sq** | **F value** | **P Value** |
| --- | --- | --- | --- | --- | --- |
| **Imputation Method** | 2 | 102.18 | 51.09 | 21877 | <2e-16 *** |
| **Percent Missing** | 2 | 6.55 | 3.27 | 1402 | <2e-16 *** |
| **Correlation Type** | 2 | 5.35 | 2.67 | 1145 | <2e-16 *** |
| **Residuals** | 2693 | 6.29 | 0.00 |  |  |

**Table S4.**

Average RMSE of 100 datasets, 20 samples by 400 metabolites for zero, minimum and mean imputation methods. Total missing was considered at 9%, 15% and 30%, and within each missing, MNAR was greater than MAR.

| **MNAR/MAR** | **DATA** | **CORR** | **Zero** | **Min** | **Mean** |
| --- | --- | --- | --- | --- | --- |
| **6% / 3%** | **DATA 1** | **POS 0.7** | 5.568 (0.164) | 5.490 (0.172) | 5.539 (0.172) |
|  | **DATA 2** | **AR(1) 0.9** | 5.561 (0.148) | 5.482 (0.152) | 5.531 (0.152) |
|  | **DATA 3** | **MIX 0.7** | 5.565 (0.159) | 5.583 (0.171) | 5.626 (0.168) |
| **10% / 5%** | **DATA 1** | **POS 0.7** | 5.118 (0.143) | 5.507 (0.137) | 5.118 (0.145) |
|  | **DATA 2** | **AR(1) 0.9** | 5.127 (0.154) | 5.066 (0.154) | 5.131 (0.154) |
|  | **DATA 3** | **MIX 0.7** | 5.240 (0.145) | 5.169 (0.143) | 5.229 (0.146) |
| **20% / 10%** | **DATA 1** | **POS 0.7** | 4.133 (0.149) | 4.224 (0.142) | 4.251 (0.156) |
|  | **DATA 2** | **AR(1) 0.9** | 4.134 (0.140) | 4.229 (0.133) | 4.251 (0.145) |
|  | **DATA 3** | **MIX 0.7** | 4.226 (0.132) | 4.306 (0.123) | 4.335 (0.130) |

**Table S5.** Average RMSE of 100 datasets, 50 samples by 400 metabolites for zero, minimum and mean imputation methods. Total missing was considered at 9%, 15% and 30%, and within each missing, MNAR was greater than MAR.

| **MNAR/MAR** | **DATA** | **CORR** | **Zero** | **Min** | **Mean** |
| --- | --- | --- | --- | --- | --- |
| **6% / 3%** | **DATA 1** | **POS 0.7** | 5.604 (0.131) | 5.520 (0.136) | 5.569 (0.138) |
|  | **DATA 2** | **AR(1) 0.9** | 5.607 (0.124) | 5.526 (0.128) | 5.570 (0.127) |
|  | **DATA 3** | **MIX 0.7** | 5.688 (0.115) | 5.602 (0.119) | 5.651 (0.119) |
| **10% / 5%** | **DATA 1** | **POS 0.7** | 5.183 (0.136) | 5.113 (0.130) | 5.176 (0.135) |
|  | **DATA 2** | **AR(1) 0.9** | 5.181 (0.127) | 5.109 (0.118) | 5.176 (0.121) |
|  | **DATA 3** | **MIX 0.7** | 5.295 (0.127) | 5.209 (0.119) | 5.279 (0.124) |
| **20% / 10%** | **DATA 1** | **POS 0.7** | 4.179 (0.144) | 4.256 (0.133) | 4.294 (0.147) |
|  | **DATA 2** | **AR(1) 0.9** | 4.182 (0.136) | 4.256 (0.126) | 4.294 (0.137) |
|  | **DATA 3** | **MIX 0.7** | 4.279 (0.135) | 4.338 (0.124) | 4.387 (0.137) |

**Table S6.** Average RMSE of 100 datasets, 100 samples by 900 metabolites for zero, minimum and mean imputation methods. Total missing was considered at 9%, 15% and 30%, and within each missing, MNAR was greater than MAR.

| **MNAR/MAR** | **DATA** | **CORR** | **Zero** | **Min** | **Mean** |
| --- | --- | --- | --- | --- | --- |
| **6% / 3%** | **DATA 1** | **POS 0.7** | 5.607 (0.083) | 5.522 (0.087) | 5.570 (0.086) |
|  | **DATA 2** | **AR(1) 0.9** | 5.608 (0.073) | 5.524 (0.075) | 5.571 (0.076) |
|  | **DATA 3** | **MIX 0.7** | 5.694 (0.085) | 5.607 (0.089) | 5.654 (0.089) |
| **10% / 5%** | **DATA 1** | **POS 0.7** | 5.194 (0.104) | 5.121 (0.097) | 5.186 (0.100) |
|  | **DATA 2** | **AR(1) 0.9** | 5.194 (0.096) | 5.110 (0.091) | 5.185 (0.092) |
|  | **DATA 3** | **MIX 0.7** | 5.311 (0.091) | 5.219 (0.084) | 5.291 (0.088) |
| **20% / 10%** | **DATA 1** | **POS 0.7** | 4.191 (0.091) | 4.266 (0.085) | 4.305 (0.089) |
|  | **DATA 2** | **AR(1) 0.9** | 4.188 (0.088) | 4.266 (0.083) | 4.303 (0.087) |
|  | **DATA 3** | **MIX 0.7** | 4.286 (0.092) | 4.345 (0.087) | 4.394 (0.094) |

**Table S7**

**a.** Specific differences in RMSE values for the KNN-CR and KNN-EU methods compared to the KNN-TR method for Myocardial dataset.

|  | **Estimate** | **Std. Error** | **t Value** | **P Value** |
| --- | --- | --- | --- | --- |
| **KNN-CR – KNN-TN** | 0.005 | 0.00275 | 1.737 | 0.0825 |
| **KNN-EU – KNN-TN** | 0.152 | 0.00275 | 55.333 | <2e-16 *** |

**b. ANOVA table giving the significance of the four factors for the Myocardial dataset.**

|  | **Df** | **Sum Sq** | **Mean Sq** | **F value** | **P Value** |
| --- | --- | --- | --- | --- | --- |
| **Imputation Method** | 2 | 17.95 | 8.98 | 1979.08 | <2e-16 *** |
| **Percent Missing** | 2 | 1.88 | 0.94 | 207.76 | <2e-16 *** |
| **Group** | 1 | 37.82 | 37.82 | 8339.65 | <2e-16 *** |
| **Sample Size** | 1 | 0.14 | 0.14 | 31.56 | 2.08e-08 |
| **Residuals** | 3593 | 16.30 | 0.00 |  |  |

**Table S8**

**a.** Specific differences in RMSE values for the KNN-CR and KNN-EU methods compared to the KNN-TR method for Atherothrombotic dataset.

|  | **Estimate** | **Std. Error** | **t Value** | **P Value** |
| --- | --- | --- | --- | --- |
| **KNN-CR – KNN-TN** | 0.019 | 0.00239 | 479.375 | <2e-16 *** |
| **KNN-EU – KNN-TN** | 0.240 | 0.00207 | 9.261 | <2e-16 *** |

**b. ANOVA table giving the significance of the four factors for the Atherothrombotic dataset.**

|  | **Df** | **Sum Sq** | **Mean Sq** | **F value** | **P Value** |
| --- | --- | --- | --- | --- | --- |
| **Imputation Method** | 2 | 64.25 | 32.13 | 8330.9 | <2e-16 *** |
| **Percent Missing** | 2 | 1.65 | 0.82 | 213.6 | <2e-16 *** |
| **Group** | 2 | 27.07 | 13.54 | 3510.5 | <2e-16 *** |
| **Sample Size** | 1 | 5.99 | 5.99 | 1554.0 | <2e-16 *** |
| **Residuals** | 5392 | 20.79 | 0.00 |  |  |

**Table S9**

**a.** Specific differences in RMSE values for the KNN-CR and KNN-EU methods compared to the KNN-TR method for African Race dataset.

|  | **Estimate** | **Std. Error** | **t Value** | **P Value** |
| --- | --- | --- | --- | --- |
| **KNN-CR – KNN-TN** | 0.015 | 0.002532 | 5.941 | 3.4e-09 *** |
| **KNN-EU – KNN-TN** | 0.087 | 0.002532 | 34.523 | <2e-16 *** |

**b. ANOVA table giving the significance of the four factors for the African Race dataset.**

|  | **Df** | **Sum Sq** | **Mean Sq** | **F value** | **P Value** |
| --- | --- | --- | --- | --- | --- |
| **Imputation Method** | 2 | 2.621 | 1.3103 | 681.4 | <2e-16 *** |
| **Percent Missing** | 2 | 3.478 | 1.7388 | 904.2 | <2e-16 *** |
| **Group** | 1 | 3.054 | 3.054 | 1588.2 | <2e-16 *** |
| **Residuals** | 1794 | 3.450 |  |  |  |

**Table S10.** Average RMSE of 100 simulations using the in vivo myocardial infarction dataset for zero, minimum and mean imputation methods. Total missing was considered at 9%, 15% and 30%, and within each missing, MNAR was greater than MAR.

| **MNAR/MAR** | **SAMPLE SIZE** | **GROUP** | **Zero** | **Min** | **Mean** |
| --- | --- | --- | --- | --- | --- |
| **6% / 3%** | **25** | **CASES** | 4.530 (0.175) | 3.454 (0.116) | 3.474 (0.117) |
|  | **25** | **CONTROLS** | 4.213 (0.213) | 3.234 (0.103) | 3.246 (0.097) |
|  | **50** | **CASES** | 4.556 (0.139) | 3.506 (0.090) | 3.528 (0.092) |
|  | **50** | **CONTROLS** | 4.256 (0.158) | 3.328 (0.069) | 3.339 (0.068) |
| **10% / 5%** | **25** | **CASES** | 4.908 (0.173) | 3.262 (0.093) | 3.297 (0.094) |
|  | **25** | **CONTROLS** | 4.704 (0.203) | 3.046 (0.082) | 3.058 (0.088) |
|  | **50** | **CASES** | 4.921 (0.142) | 3.282 (0.087) | 3.315 (0.089) |
|  | **50** | **CONTROLS** | 4.742 (0.124) | 3.098 (0.053) | 3.118 (0.053) |
| **20% / 10%** | **25** | **CASES** | 6.053 (0.169) | 2.091 (0.076) | 2.949 (0.077) |
|  | **25** | **CONTROLS** | 5.879 (0.158) | 2.796 (0.051) | 2.803 (0.053) |
|  | **50** | **CASES** | 6.050 (0.117) | 2.916 (0.057) | 2.960 (0.056) |
|  | **50** | **CONTROLS** | 5.900 (0.112) | 2.821 (0.037) | 2.842 (0.040) |

**Table S11.** Average RMSE of 100 simulations using the human atherothrombotic dataset for zero, minimum and mean imputation methods. Total missing was considered at 9%, 15% and 30%, and within each missing, MNAR was greater than MAR.

| **MNAR/MAR** | **SAMPLE SIZE** | **GROUP** | **Zero** | **Min** | **Mean** |
| --- | --- | --- | --- | --- | --- |
| **6% / 3%** | **50** | **sCAD** | 5.437 (0.084) | 4.331 (0.066) | 4.363 (0.067) |
|  | **50** | **TYPE1** | 5.608 (0.087) | 4.543 (0.080) | 4.580 (0.082) |
|  | **50** | **TYPE2** | 5.652 (0.093) | 4.547 (0.094) | 4.588 (0.093) |
|  | **100** | **sCAD** | 5.429 (0.053) | 4.329 (0.045) | 4.362 (0.046) |
|  | **100** | **TYPE1** | 5.569 (0.057) | 4.463 (0.058) | 4.500 (0.059) |
|  | **100** | **TYPE2** | 5.629 (0.062) | 4.504 (0.061) | 5.542 (0.062) |
| **10% / 5%** | **50** | **sCAD** | 5.876 (0.081) | 4.066 (0.051) | 4.117 (0.052) |
|  | **50** | **TYPE1** | 5.988 (0.083) | 4.188 (0.073) | 4.245 (0.077) |
|  | **50** | **TYPE2** | 6.056 (0.070) | 4.216 (0.066) | 4.273 (0.068) |
|  | **100** | **sCAD** | 5.891 (0.052) | 4.079 (0.035) | 4.131 (0.036) |
|  | **100** | **TYPE1** | 6.013 (0.055) | 4.216 (0.056) | 4.270 (0.059) |
|  | **100** | **TYPE2** | 6.066 (0.051) | 4.216 (0.056) | 4.273 (0.057) |
| **20% / 10%** | **50** | **sCAD** | 7.001 (0.064) | 3.519 (0.041) | 3.581 (0.042) |
|  | **50** | **TYPE1** | 7.141 (0.070) | 3.613 (0.052) | 3.686 (0.056) |
|  | **50** | **TYPE2** | 7.206 (0.070) | 3.716 (0.052) | 3.801 (0.057) |
|  | **100** | **sCAD** | 7.019 (0.048) | 3.550 (0.032) | 3.612 (0.034) |
|  | **100** | **TYPE1** | 7.152 (0.050) | 3.638 (0.046) | 3.711 (0.050) |
|  | **100** | **TYPE2** | 7.207 (0.043) | 3.705 (0.041) | 3.786 (0.046) |

**Table S12.** Average RMSE of 100 simulations using the African Race dataset for zero, minimum and mean imputation methods. Total missing was considered at 9%, 15% and 30%, and within each missing, MNAR was greater than MAR.

| **MNAR/MAR** | **SAMPLE SIZE** | **GROUP** | **Zero** | **Min** | **Mean** |
| --- | --- | --- | --- | --- | --- |
| **6% / 3%** | **15** | **Tanzania** | 4.114 (0.167) | 2.752 (0.078) | 2.764 (0.078) |
|  | **25** | **Ethiopia** | 4.022 (0.131) | 2.673 (0.055) | 2.685 (0.053) |
| **10% / 5%** | **15** | **Tanzania** | 4.567 (0.173) | 2.570 (0.074) | 2.579 (0.075) |
|  | **25** | **Ethiopia** | 4.535 (0.114) | 2.489 (0.048) | 2.499 (0.050) |
| **20% / 10%** | **15** | **Tanzania** | 5.862 (0.119) | 2.293 (0.052) | 2.288 (0.057) |
|  | **25** | **Ethiopia** | 5.813 (0.108) | 2.255 (0.035) | 2.249 (0.037) |

**Table S13.** Specific differences in MLCI values for the Zero, minimum, mean, KNN-CR and KNN-EU methods compared to the KNN-TR method for the Myocardial Infarction data.

| **Contrast** | **Estimate** | **Std. Error** | **t Value** | **P Value** |
| --- | --- | --- | --- | --- |
| **Zero – KNN-TN** | -0.511 | 0.0046 | -96.82 | <2e-16 *** |
| **Min – KNN-TN** | -0.152 | 0.00527 | -28.75 | <2e-16 *** |
| **Mean – KNN-TN** | -0.038 | 0.00527 | -7.23 | 5.8e-13 |
| **KNN-CR – KNN-TN** | -0.002 | 0.00527 | -0.41 | 0.679 |
| **KNN-EU – KNN-TN** | -0.024 | 0.00527 | -4.42 | 1.03e-05 |

**b. ANOVA table giving the significance of the three factors in the simulation study for Myocardial Infarction data.**

|  | **Df** | **Sum Sq** | **Mean Sq** | **F value** | **P Value** |
| --- | --- | --- | --- | --- | --- |
| **Imputation Method** | 5 | 118.71 | 23.742 | 2846.5 | <2e-16 *** |
| **Percent Missing** | 2 | 8.81 | 4.407 | 528.4 | <2e-16 *** |
| **Sample Size** | 1 | 3.18 | 3.178 | 381.0 | <2e-16 *** |
| **Residuals** | 3591 | 29.95 | 0.008 |  |  |

**Table S14.** Specific differences in MLCI values for the Zero, minimum, mean, KNN-CR and KNN-EU methods compared to the KNN-TR method for the Atherothrombotic data.

| **Contrast** | **Estimate** | **Std. Error** | **t Value** | **P Value** |
| --- | --- | --- | --- | --- |
| **Zero – KNN-TN** | -0.295 | 0.0044 | .-58.47 | <2e-16 *** |
| **Min – KNN-TN** | -0.112 | 0.0050 | -22.12 | <2e-16 *** |
| **Mean – KNN-TN** | -0.036 | 0.0050 | -7.17 | 8.9e-13 |
| **KNN-CR – KNN-TN** | -0.001 | 0.0050 | 0.25 | 0.803 |
| **KNN-EU – KNN-TN** | -0.017 | 0.0050 | -3.36 | 0.0008 |

**b. ANOVA table giving the significance of the three factors in the simulation study for Atherothrombotic data.**

|  | **Df** | **Sum Sq** | **Mean Sq** | **F value** | **P Value** |
| --- | --- | --- | --- | --- | --- |
| **Imputation Method** | 5 | 39.67 | 7.93 | 1037.9 | <2e-16 *** |
| **Percent Missing** | 2 | 2.08 | 1.04 | 136.1 | <2e-16 *** |
| **Sample Size** | 1 | 3.10 | 3.10 | 405.0 | <2e-16 *** |
| **Residuals** | 3591 | 27.45 | 0.01 |  |  |

**Table S15.** Specific differences in MLCI values for the Zero, minimum, mean, KNN-CR and KNN-EU methods compared to the KNN-TR method for the African Race data.

| **Contrast** | **Estimate** | **Std. Error** | **t Value** | **P Value** |
| --- | --- | --- | --- | --- |
| **Zero – KNN-TN** | -0.239 | 0.0080 | -24.523 | <2e-16 *** |
| **Min – KNN-TN** | -0.139 | 0.0097 | -14.214 | <2e-16 *** |
| **Mean – KNN-TN** | -0.008 | 0.0097 | -0.849 | 0.396 |
| **KNN-CR – KNN-TN** | -0.001 | 0.0097 | 0.052 | 0.958 |
| **KNN-EU – KNN-TN** | -0.003 | 0.0097 | -0.262 | 0.795 |

**b. ANOVA table giving the significance of the three factors in the simulation study for Myocardial Infarction data.**

|  | **Df** | **Sum Sq** | **Mean Sq** | **F value** | **P Value** |
| --- | --- | --- | --- | --- | --- |
| **Imputation Method** | 5 | 15.39 | 3.079 | 216.1 | <2e-16 *** |
| **Percent Missing** | 2 | 1.06 | 0.530 | 37.2 | <2e-16 *** |
| **Residuals** | 1792 | 25.53 | 0.014 |  |  |
